# Correlations Between Patient-Reported Dysphagia Screening and Penetration–Aspiration Scores in Head and Neck Cancer Patients Post-oncological Treatment

**DOI:** 10.1007/s00455-017-9847-6

**Published:** 2017-09-08

**Authors:** Johanna Hedström, Lisa Tuomi, Caterina Finizia, Caroline Olsson

**Affiliations:** 1 Department of Otorhinolaryngology, Head and Neck Surgery, Sahlgrenska University Hospital, Institute of Clinical Sciences, Sahlgrenska Academy at the University of Gothenburg, Gothenburg, Sweden; 2 Department of Radiation Physics, Institute of Clinical Sciences, The Sahlgrenska Academy at the University of Gothenburg, Gothenburg, Sweden; 3 Regional Cancer Center West, The Western Sweden Healthcare Region, Gothenburg, Sweden

**Keywords:** Head and neck neoplasms, Deglutition, Deglutition disorders, Penetration–aspiration scale (PAS), Patient-reported outcomes (PRO), Radiotherapy, Chemoradiotherapy

## Abstract

Dysphagia is a common and severe toxicity after oncological treatment of head and neck cancer (HNC). The study aim was to investigate relationships between patient-reported dysphagia and clinically measured swallowing function in HNC after modern curative radiotherapy with or without chemotherapy to identify possible alarm symptoms for clinically manifest dysphagia. Patients with tumors of the tonsil, base of tongue, hypopharynx, and larynx treated in 2007–2015 were assessed for dysphagia post-treatment by telephone interview and videofluoroscopy (VFS). A study-specific categorized symptom score was used to determine patient-reported dysphagia (DESdC = presence of Drinking, Eating, Swallowing difficulties, and Coughing when eating/drinking (any combination); scores between 0 and 4 with 0 = no symptom); the penetration–aspiration scale (PAS) to determine swallowing function by VFS. Swallowing difficulties were defined as DESdC ≥ 1 and PAS ≥ 2. Relationships between clinically relevant cut-offs for DESdC and PAS were determined by Pearson’s correlation coefficient (Pr). Swallowing difficulties according to DESdC were reported by 89% of the patients and according to PAS by 60% at a median of 7 months post-treatment. Averaged correlations between DESdC score 1/2/3/4 and PAS were 0.16/0.10/0.27/0.18. Almost one in two patients with DESdC score ≥3 had severe swallowing difficulties according to PAS. Correlations between individual DESdC:s were highest for swallowing and eating (Pr = 0.53) and lowest for swallowing and coughing (Pr = 0.11). Our data suggest that if a patient reports having swallowing difficulties, it is likely that he or she also has eating difficulties but not necessarily coughing problems when eating/drinking. However, if all these three symptoms are reported, it is likely that the patient will present with moderate or severe impaired swallowing function according to PAS and thus should be referred for further evaluation and treatment.

## Introduction

Dysphagia is the most common long-term side effect of radiation therapy (RT) for head and neck cancer (HNC) [1]. Radiation-induced dysphagia affects more than 50% of HNC patients [2], making it a dose-limiting toxicity [3]. Swallowing difficulties are associated with high risk of aspiration pneumonia, malnutrition, and dehydration [4]. The patients’ general health as well as quality of life (QoL) is affected [5, 6]. Maintaining swallowing ability is, therefore, of great importance [1]. In the light of this, research is focused on finding predictors of post-treatment dysphagia, developing treatment regimens with reduced toxicity, and preventive swallowing interventions. However, currently, recommendations for screening questions to detect dysphagia in the daily patient-doctor consultation are missing.

Identifying a comprehensive measure of dysphagia is challenging due to the complexity of swallowing physiology. A common approach is to use videofluoroscopy (VFS), where airway protection is evaluated and associated swallowing function is scored according to the penetration–aspiration scale (PAS) [7, 8] or evaluated by other measures. It is, however, important to evaluate both clinically measured swallowing function as well as the patient’s perception of swallowing [9, 10]. Patient-reported information on dysphagia can be collected by validated HNC-specific questionnaires, e.g., the M D Anderson dysphagia inventory (MDADI) [11] and the European Organization of Research and Treatment of Cancer, Quality of Life Questionnaire, Head and Neck 35 (EORTC QLQ H&N35) [12]. These are, however, typically very extensive and more applicable in clinical research studies than in the everyday doctor-patient encounters. Also, clinical measures and patient-reported outcomes (PRO) regarding dysphagia generally correlate poorly [9, 10, 13, 14]. Having an easily accessible and reliable screening tool based on patient-reported dysphagia, that indicates if the patient needs further evaluation or treatment, would be of great use in clinical practice.

The purpose of this work was to investigate relationships between four dysphagia-specific questions and clinically measured swallowing function in HNC after modern curative radiotherapy with or without chemotherapy, in order to identify possible alarm symptoms for clinically manifest dysphagia. We used information from 118 patients who had been assessed for dysphagia by telephone interview post-treatment and by VFS. Correlations between these two approaches were made to identify the most useful question, or combination of questions, to serve as a screening tool for clinically manifest dysphagia.

## Subjects and Methods

### Subjects

Patients with newly diagnosed HNC presented at the weekly multidisciplinary tumor board meeting at Sahlgrenska University Hospital Gothenburg Sweden were identified as potential study participants. If the patients reported swallowing problems by telephone interview at least 6 months after oncological treatment, they were included in a prospective study. This work concerns patients recruited between November 2010 and June 2016. The patients were treated in 2007–2015 with external beam RT only or in combination with brachytherapy, with or without chemotherapy, but not with surgery.

The inclusion criteria were for patients to be diagnosed with cancers of the tonsil, base of tongue, hypopharynx or larynx, treated with curative oncological treatment as described above, and having undergone VFS 6–36 months post-oncological treatment. Patients who declined VFS examination, who were planned for palliative treatment, or who experienced dysphagia before cancer diagnosis were not eligible for inclusion. Tumors were staged according to the TNM classification of tumors [15].

The follow-up involved swallowing function as scored by the penetration–aspiration scale (PAS) [8] on VFS, as well as PRO information on dysphagia (questions regarding drinking, eating and swallowing difficulties, and coughing when eating/drinking). Comorbidity was evaluated according to the Adult Comorbidity Evaluation 27 (ACE-27) [16].

### Treatment Information

External beam RT was planned based on computed tomography (CT) imaging and delivered as 3D-conformal radiation therapy (3D-CRT) or intensity-modulated/volumetric-modulated radiation therapy (IMRT/VMAT). The Eclipse™ treatment planning system was used for treatment planning (versions 8.1, 8.6, 8.9, 10.0, and 11.0, Varian Medical Systems, Palo Alto, U.S.). Prescribed doses were typically in the range 65–68 Gy with 1.7–2.0 Gy/fraction once or twice daily, five days a week. The dose was prescribed according to the principles of the International Commission on Radiation Units and Measurements (ICRU) [17, 18].

Brachytherapy was, when applicable, given after completed external beam RT according to local guidelines. Orthogonal X-ray imaging was used for treatment planning in the Brachy Vision module of Eclipse™. Pulsed-dose rate (PDR) brachytherapy (^192^Ir-source) was delivered every two hours (dose per pulse: of 1.3–1.4 Gy), five times a day, until the prescribed dose of 11 Gy was reached.

Chemotherapy was given as either induction or concomitant therapy. Induction chemotherapy generally consisted of two cycles of cisplatin, 100 mg/m^2^ day one and 5-Fu (Fluoracil) 1000 mg/m^2^ per day by continuous infusion day one through five. The cycle interval was 22 days. Concomitant chemotherapy generally consisted of six cycles of cisplatin, 40 mg/m^2^ day one, with a cycle interval of seven days.

### Dysphagia Assessment and Endpoints

#### Patient-Reported Outcome Information

The PRO information was collected by a semi-structured telephone interview. The interviews were conducted by five speech-language pathologists (SLP), following written guidelines. All patients were asked four questions regarding swallowing disability. Do you have difficulties: (1) drinking? (2) eating? (3) swallowing? (4) Do you cough when eating/drinking? The answers were documented as yes or no. From these questions, a study-specific categorized symptom score was constructed, DESdC (acronym for Drinking, Eating, Swallowing difficulties, and Coughing when eating/drinking), describing the presence of any combination of these symptoms. We also constructed a DESdC score, ranging from 0 to 4; 0 = no to all questions; 1 = yes to one question; 2 = yes to two questions; 3 = yes to three questions; 4 = yes to all four questions.

#### Videofluoroscopy and Objective Scoring of Swallowing

**Fig. 1:**
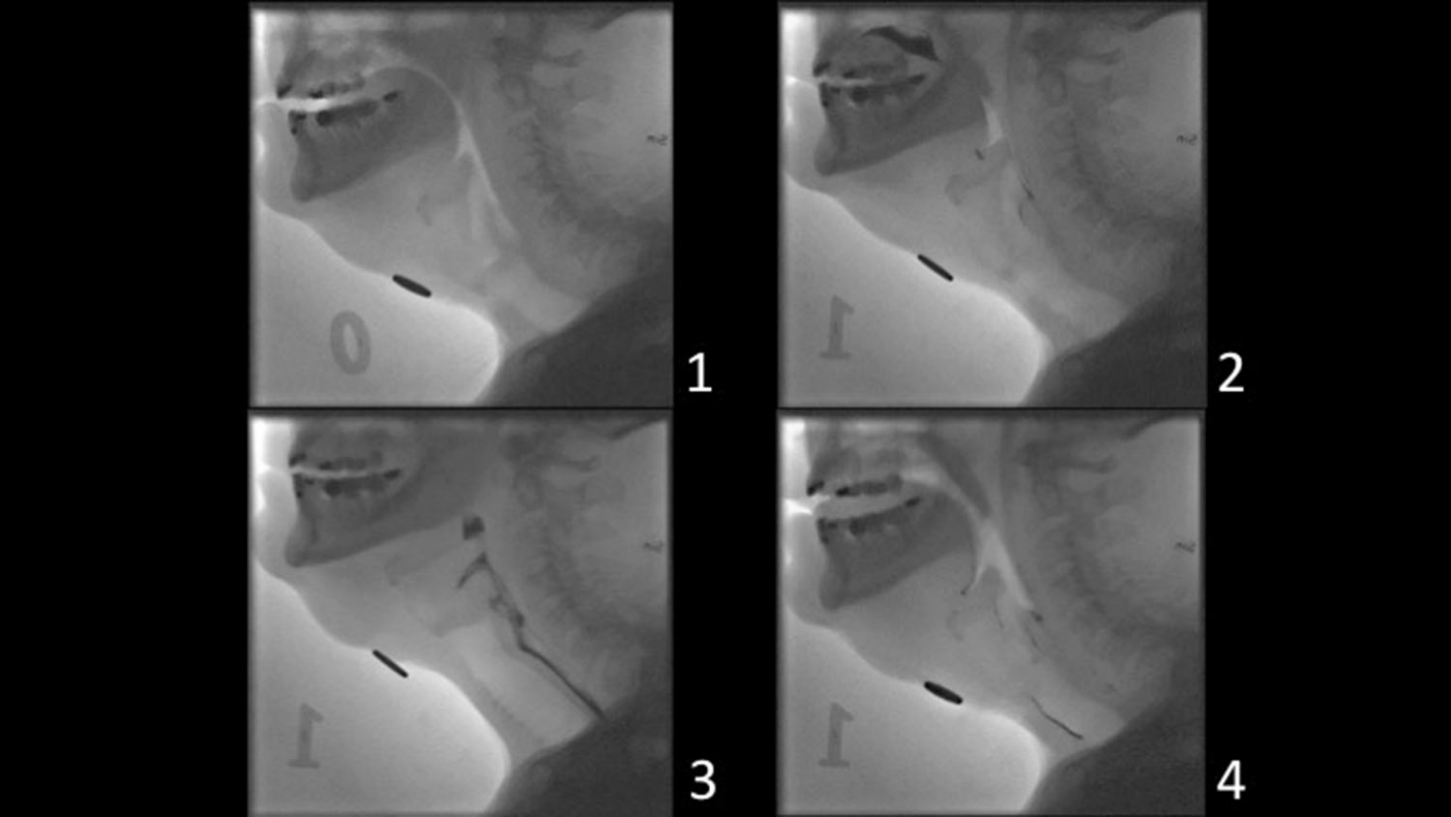
Swallowing with bolus aspiration as visualized by videofluoroscopy (static images in lateral projection). **1** Before start of the examination. **2** The bolus (*black*) is seen in the oral cavity with residue from previous swallows in the vallecula and posterior commissure. **3** The bolus (*black*) is transported through the pharynx and into the esophagus. **4** Residue of the bolus is seen in the larynx, around the vocal folds and in the trachea

VFS examination of the swallowing function was performed with the patient in an upright position. High-resolution images (video matrix 1024 × 1024) were acquired in lateral projection at a rate of 15 frames per second and digitally stored. The field of view included the tip of the tongue anteriorly, the pharyngeal wall posteriorly, the soft palate superiorly, and the seventh cervical vertebra inferiorly (Fig. 1). Gastrointestinal radiologists trained in functional assessment of swallowing performed the examinations together with a SLP. Six boluses were observed; 3, 5, 10, and 20 ml of thin barium contrast liquid and 5 ml of a mildly thick iodine contrast consistency and 3 ml extremely thick iodine contrast consistency [categorized according to The International Dysphagia Diet Standardization Initiative (IDDSI) [19] (Table 1)]. All bolus volumes were measured by syringe and placed into the patient’s mouth via the syringe or a spoon. For all boluses, except for 20 ml thin barium, the patient was instructed to hold the bolus in his/her mouth until directed to swallow. For the 20 ml thin liquid, the patients were instructed to drink freely from a cup at a pace of their own choice. The patients were instructed to sip water to clear their pharynx between swallows. Swallowing of each bolus was performed twice. Not all patients were able to complete two swallowing attempts of each bolus. A patient may have refused to attempt one or both trials of a bolus; the SLP also may have judged it as too great a clinical risk of excessive aspiration to make a second swallowing attempt of the bolus during the VFS. Thus, for the safety of the patient, if the patient demonstrated a high degree of aspiration (e.g., PAS 7–8) on the first swallowing attempt of the bolus no second attempt was made.

**Table 1:** Detailed description of the boluses used in the present study. Each consistency is also described with the standardized terminology according to the International Dysphagia Diet Standardisation Initiative (IDDSI) [19]

Bolus number	Bolus size and consistency (level according to the IDDSI framework [19])	Contrast
1	3 ml thin liquid (0)	Mixobar Colon 1 g Barium/ml mixed with equal amount of water
2	5 ml thin liquid (0)	
3	10 ml thin liquid (0)	
4	20 ml thin liquid, drink freely (0)	
5	5 ml mildly thick (2)	Omnipaque 300 mg Iodine/ml; 20 ml Omnipaque mixed with 2 ml instant thickener
6	3 ml extremely thick (4)	Omnipaque 300 mg Iodine/ml; 20 ml Omnipaque mixed with 15 ml instant chocolate pudding mix

Two highly experienced gastrointestinal radiologists were involved in the analysis of the VFS according to Rosenbek’s PAS (Table 2) [8]. The radiologist and the SLP made a joint assessment of the worst PAS score as the VFS examination was performed. This is a clinical practice at the Sahlgrenska University Hospital. The digital technique used in the VFS examinations allowed for detailed evaluation of swallowing by both slow motion analysis as well as by static image assessment. PAS is an equal-appearing interval scale used to describe penetration and aspiration events, ranging from 1 (no material enters the airway) to 8 (material enters the airway, passes below the vocal folds and no effort is made to eject it). PAS has successfully been used to differentiate between normal and abnormal airway protection following treatment for HNC, with abnormal airway protection defined as PAS ≥ 2 [7, 9, 20–22]. PAS was therefore chosen as the single VFS outcome measure in this study. The worst overall PAS score for each patient, regardless of bolus consistency or swallowing attempt, was included in the statistical analysis.

**Table 2:** Rosenbek’s penetration–aspiration scale [8]

PAS score	Definition
1	Material does not enter the airway
2	Material enters the airway, remains above the vocal folds, and is ejected from the airway
3	Material enters the airway, remains above the vocal folds, and is not ejected from the airway
4	Material enters the airway, contacts the vocal folds, and is ejected from the airway
5	Material enters the airway, contacts the vocal folds, and is not ejected from the airway
6	Material enters the airway, passes below the vocal folds, and is ejected into the larynx or out of the airway
7	Material enters the airway, passes below the vocal folds, and is not ejected from the trachea despite effort
8	Material enters the airway, passes below the vocal folds, and no effort is made to eject

### Statistics

Descriptive statistics were used to summarize the demographical and clinical characteristics of the study subjects. The distribution of the variables was given as mean, standard deviation (SD), median and range for continuous variables, and as numbers and percentages for categorical variables.

Correlations between DESdC and PAS were calculated using Pearson’s correlation coefficients (Pr). Correlations in the range ≤0.39 were regarded as weak; 0.4–0.59 as moderate; ≥0.6 as strong [23]. DESdC was investigated for four cut-offs scores: ≥1, ≥2, ≥3, and 4. PAS was investigated for seven cut-offs: PAS ≥ 2, PAS ≥ 3, PAS ≥ 4, PAS ≥ 5, PAS ≥ 6, PAS ≥ 7, and PAS = 8 but also for three groups in accordance with symptom severity as suggested by Rosenbek et al. [8]: PAS 2 + 3, PAS 4 + 5, and PAS 6–8. Correlations between the four individual DESdC variables were also calculated using Pearson’s correlation coefficients (6 combinations). All calculations were performed in Excel (PEARSON function, Microsoft Office Excel 2016).

### Ethical Considerations

The study was conducted in accordance with the Declaration of Helsinki and was approved by the Regional Ethical Review Board in Gothenburg, Sweden. All participants gave their written informed consent before inclusion in the study.

## Results

In this study, 118 HNC patients were included, 80 men (68%) and 38 females (32%). Their median age was 62 years (range 41–88). We had complete outcome information on clinical swallowing function (PAS) on all study participants, but DESdC data were missing for two individuals. Patient characteristics and treatment information are listed in Table 3. At RT start, 14 patients (12%) reported dysphagia according to DESdC ≥ 1. The patients were, on average, assessed at 7 months after completed oncological treatment (range 6–36 months). Tumor of the tonsil was the most common (53% of patients), followed by larynx and base of tongue (20% of patients, respectively). A majority had stage four disease at the time of diagnosis (60%), 70% had nodular engagement, and 49% had mild or moderate comorbidity according to ACE-27. The majority of the patients (74%) received chemoradiotherapy.

**Table 3:** Patient characteristics and treatment information

Characteristic	*N* = 118
Age in years at RT start median (range)	62 (41–88)
BMI at RT start mean (SD)	26.1 (4.4)
	*n* (%)
Gender	
Male	80 (68)
Female	38 (32)
Smoking	
Never smoked	32 (27)
Current smoker	35 (30)
Former smoker, stopped >12 months before RT	51 (43)
Missing data	0
Comorbidity according to ACE-27 at RT start	
None (grade 0)	51 (43)
Mild (grade 1)	38 (32)
Moderate (grade 2)	20 (17)
Severe (grade 3)	10 (8)
Missing data	0
Tumor location (tumor code)	
Tonsil (C09)	63 (53)
Base of tongue (C01.9)	23 (20)
Larynx (C32.0, C32.1)	24 (20)
Hypopharynx (C12, C13)	8 (7)
Overall tumor stage	
1	16 (14)
2	12 (10)
3	19 (16)
4	71 (60)
TNM-T-stage	
I	29 (25)
II	46 (39)
III	26 (22)
IV	16 (14)
Nodular engagement	
Yes	83 (70)
No	35 (30)
Oncological treatment	
RT	31 (26)
RT+ chemotherapy	87 (74)

*ACE-27* adult comorbidity evaluation-27, *BMI* body mass index, *RT* radiotherapy, *TNM* tumor location, nodular engagement, metastasis

### Distribution of DESdC and PAS

At follow-up, 71/118 (60%) of the patients had swallowing difficulties according to PAS (PAS ≥ 2) and 103/116 (89%) reported dysphagia according to the DESdC criteria (DESdC ≥ 1; Table 4). The most commonly reported DESdC score was 3 (38/116; 33%). Among these patients, 71% (27/38) had swallowing difficulties according to the PAS criteria; 19/27 (70%) had PAS ≥ 4 and 15/27 (56%) had PAS ≥ 6.

**Table 4:** Outcome information at a median assessment time of 7 months

Endpoint	*N* = 118
PAS	*n* (%)
1	45 (39)
2	23 (20)
3	4 (3)
4	10 (9)
5	4 (3)
6	6 (5)
7	15 (13)
8	9 (8)
Patient-reported DESdC	Yes (%)
Drinking (D)	25 (22)
Eating (E)	80 (69)
Swallowing (S)	88 (76)
Coughing when eating/drinking (C)	59 (51)
	*n* (%)
DESdC total score *N* = 116	
0	13 (11)
1	22 (19)
2	28 (24)
3	38 (33)
4	15 (13)
DESdC singles *N* = 21^a^	
D	0 (0)
E	3 (14)
S	10 (48)
C	8 (38)
DESdC paired *N* = 24^b^	
D, E	0 (0)
D, S	1 (4)
D, C	0 (0)
E, S	18 (75)
E, C	2 (8)
S, C	3 (13)
DESdC triplets *N* = 38	
D, E, S	7 (19)
E, S, C	29 (76)
D, E, C	2 (5)
D, S, C	0 (0)
DESdC all four *N* = 15	
D, E, S, C	15 (100)

*DESdC* Drinking, Eating, Swallowing difficulties and Coughing when eating/drinking, *D* drinking difficulties; *E* eating difficulties, *S* swallowing difficulties, *C* coughing when eating/drinking, *PAS* penetration–aspiration scale

^a^Of the 22 patients reporting one symptom there was one individual who had missing data (not answering all four questions)

^b^Of the 28 patients reporting two symptoms there were four individuals who had missing data (not answering all four questions)

**Fig. 2:**
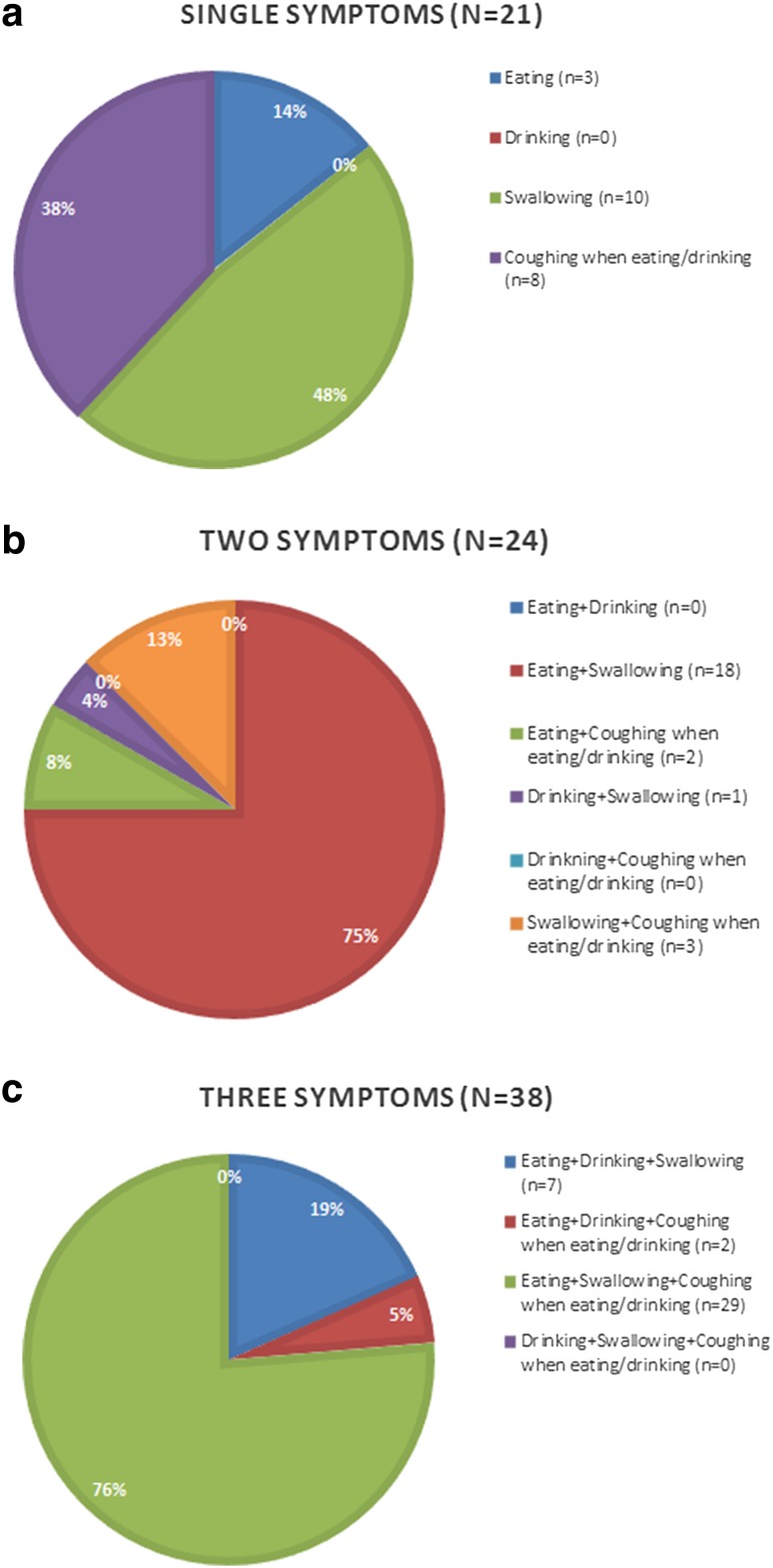
Distribution of single and multiple DESdC

There are fifteen possible DESdC symptom combinations and fifteen patients reported all four symptoms (Table 4; Fig. 2). Among the patients reporting one symptom (*n* = 21), almost one in two patients reported swallowing difficulties (10/21), whereas no one reported difficulties in drinking. In the group reporting two symptoms (*n* = 24), three of four reported eating and swallowing difficulties (18/24), but no one reported the combinations of neither difficulties in eating and drinking nor difficulties in drinking and coughing when eating/drinking. Last, the combination of eating and swallowing difficulties and coughing when eating/drinking was the most commonly reported combination among the 38 patients reporting three symptoms (76%). Here, no one reported the combination of drinking and swallowing difficulties together with coughing when eating/drinking.

Regarding correlations within individual DESdC (Table 5), the highest correlation (Pr = 0.53) was between “Do you have difficulties swallowing?” and “Do you have difficulties eating?”; the lowest for “Do you have difficulties swallowing?” and “Do you cough when eating/drinking?” (Pr = 0.11).

**Table 5:** Correlations between individual DESdC

DESdC	Pearson’s Pr
Eating vs drinking	0.32
Eating vs swallowing	**0.53**
Eating vs coughing when eating/drinking	0.29
Drinking vs swallowing	0.21
Drinking vs coughing when eating/drinking	0.16
Swallowing vs coughing when eating/drinking	0.11

The highest correlations marked in bold

*DESdC* Drinking, Eating, Swallowing difficulties and Coughing when eating/drinking, *Pr* correlation coefficient

### Correlations Between DESdC and PAS

DESdC ≥ 3 presented with higher correlation coefficients regardless of PAS (Pr ≤ 0.33; Table 6). When grouping PAS scores according to symptom severity, as suggested by Rosenbek et al. [8], the highest correlation coefficient was found for DESdC ≥ 3 and PAS 6–8 (Table 7). Almost 50% of the patients with DESdC score ≥ 3 had severe swallowing difficulties according to PAS (PAS ≥ 6). Of the patients with DESdC = 3 (*n* = 38), three of four had problems eating, swallowing, and coughing when eating/drinking (29/38).

**Table 6:** Correlations between DESdC and PAS

PAS	DESdC	Pearson’s Pr	Pr mean	Pr std	Pr median	Pr min	Pr max
1–7	0–4	0.30					
2+	1+	0.11	0.19	0.10	0.18	0.09	0.30
	2+	0.09					
	3+	**0.30**					
	4	0.25					
3+	1+	0.24	0.19	0.09	0.19	0.09	0.28
	2+	0.09					
	3+	**0.28**					
	4	0.15					
4+	1+	0.22	0.20	0.07	**0.20**	0.13	0.28
	2+	0.13					
	3+	**0.28**					
	4	0.18					
5+	1+	0.17	0.18	0.07	0.16	0.13	0.28
	2+	0.13					
	3+	**0.28**					
	4	0.15					
6+	1+	0.15	**0.21**	0.08	0.18	0.15	**0.33**
	2+	0.17					
	3+	**0.33**					
	4	0.18					
7+	1+	0.11	0.18	0.09	0.15	0.10	0.30
	2+	0.10					
	3+	**0.30**					
	4	0.18					
8	1+	0.10	0.10	0.08	0.11	−0.02	0.18
	2+	−0.02					
	3+	0.12					
	4	**0.18**					

The highest correlations marked in bold

*DESdC* Drinking, Eating, Swallowing difficulties and Coughing when eating/drinking, *1* score 1, *2* score 2, *3* score 3, *4* score 4, *PAS* penetration–aspiration scale, *Pr* correlation coefficient

**Table 7:** Correlation between DESdC and PAS grouped in line with Rosenbek et al. [8]

PAS	DESd	Pearson’s Pr
1–4	0–4	0.29
2–3	1	−0.06
	2	−0.07
	3	−0.04
	4	**0.09**
4–5	1	**0.16**
	2	−0.02
	3	−0.03
	4	0.01
6–8	1	−0.08
	2	−0.20
	3	**0.22**
	4	0.18

The highest correlations marked in bold

*DESdC* Drinking, Eating, Swallowing difficulties and Coughing when eating/drinking, *1* score1, *2* score 2, *3* score 3, *4* score 4, *PAS* penetration–aspiration scale, *Pr* correlation coefficient

When stratifying for assessment time, a median split at 7 months resulted in two groups with median assessment times of 6.5 and 12 months, respectively, and affected the correlations between DESdC and PAS (Table 8). For shorter assessment time, Pr reduced with approximately 0.1 with respect to non-stratified results; for longer assessment time, Pr typically increased with 0.1. A similar trend was also seen in the grouped analyses (data not shown).

**Table 8:** Correlation between DESdC and PAS, adjusted for assessment time

PAS	DESdC	Pearson’s Pr
≤7 months		
1–7	0–4	0.14
2+	1+	0.19
	2+	0.13
	3+	**0.26**
	4	0.21
3+	1+	**0.17**
	2+	−0.03
	3+	0.12
	4	−0.01
4+	1+	**0.16**
	2+	0.03
	3+	**0.16**
	4	0.00
5+	1+	0.12
	2+	0.13
	3+	**0.21**
	4	−0.09
6+	1+	0.09
	2+	0.08
	3+	**0.22**
	4	−0.06
7+	1+	0.06
	2+	0.03
	3+	**0.14**
	4	−0.03
8	1+	0.09
	2+	−0.20
	3+	−0.08
	4	−0.09
>7 months		
1–7	0–4	0.45
2+	1+	0.01
	2+	0.07
	3+	**0.38**
	4	0.28
3+	1+	0.31
	2+	0.20
	3+	**0.45**
	4	0.26
4+	1+	0.28
	2+	0.21
	3+	**0.40**
	4	0.30
5+	1+	0.22
	2+	0.14
	3+	**0.36**
	4	0.32
6+	1+	0.20
	2+	0.25
	3+	**0.43**
	4	0.36
7+	1+	0.17
	2+	0.17
	3+	**0.45**
	4	0.35
8	1+	0.11
	2+	0.10
	3+	0.27
	4	**0.33**

The highest correlations marked in bold

*DESdC* Drinking, Eating, Swallowing difficulties and Coughing when eating/drinking, *1* grade 1, *2* grade 2, *3* grade 3, *4* grade 4, *PAS* penetration–aspiration scale, *Pr* correlation coefficient

## Discussion

In this study, we explored correlations between PRO information on dysphagia and clinical outcome measures regarding swallowing impairment following nonsurgical treatment of HNC. We found the highest correlations between three or more patient-reported symptoms and PAS in HNC patients 7 months after oncological treatment. Among the patients reporting this, almost one in two patients had severe swallowing difficulties (PAS ≥ 6). Two thirds of difficulties were reported as problems with eating, swallowing, and coughing when eating/drinking. Difficulties in drinking were rarely reported by the patients for any combination of DESdC symptoms.

Our results add to previous findings by clarifying the relationships between individual questions or combinations of questions on dysphagia and clinically manifest dysphagia. Although the current study is one of few studies to look at independent question items instead of complete questionnaire results, our findings are in line with previous research which shows that clinical measurements and PRO information regarding dysphagia generally correlate poorly [9, 10, 13, 24]. One example is the study by Van der Molen et al. [10], where statistically non-significant correlations between PAS and the patients’ perceived swallowing ability were found. Cut-offs for meaningful correlations was, as in our study, taken as 0.3. Three questions from their study-specific questionnaire also correspond to our questions on swallowing and drinking difficulties. Another example where no significant correlations were found is the prospective study by Rogus-Pulia et al. [24]. They used a modification of a previous questionnaire by Logemann et al. [25] and two questions reflected our questions on swallowing difficulties and coughing when eating/drinking.

Considering this general discrepancy between the patients’ perception of swallowing and clinically measured swallowing function, reflected in weak correlations, there are situations where the patients perceive normal swallowing but the clinical examination shows severe swallowing dysfunction and vice versa [10]. On the other hand, the study by Boczko et al. [26] on a geriatric cohort highlights that the patients’ awareness of their swallowing function represents an important aspect of functional recovery although they are less discriminating than clinicians in recognizing swallowing impairment. This was also shown by Rogus-Pulia et al. [24]. Furthermore, the study by Pauloski et al. [27] suggests that complaints of dysphagia may act as a reliable indicator of aspiration. Our data support this very important aspect. We found that the large majority of the patients with severe swallowing dysfunction according to PAS (73%), with high risk of aspiration pneumonia as a consequence, reported at least three dysphagia-related symptoms. One probable explanation for fewer patients reporting four symptoms (13%) than three symptoms (33%) is the occurrence of silent aspiration. Therefore, the symptom “coughing when eating/drinking” could be an alarm symptom for silent aspiration, in particular if it is reported as a single symptom. The study by Rogus-Pulia et al. [24] showed that, in their patient cohort, all occurrences of penetration and 83% of aspiration occurrences were “silent”. They also showed that higher amounts of pharyngeal residue were found post-treatment compared to pre-treatment, but the patients did not report higher occurrence of food sticking in the throat. For that reason, patients are not always aware of all dysphagia-related symptoms and accordingly do not report them.

The strengths of this study are the use of consecutively recruited patients and the relatively large cohort. To ensure structured collection of patient-reported data and to minimize interviewer-related bias, we developed written guidelines for the telephone interviews. A limitation of the study is the development and use of a study-specific categorized symptom score as opposed to using a validated questionnaire. However, effects by individual items in commonly used questionnaires may be hard to identify since items typically are to be summarized according to certain strategies. Using individual questions was of importance for the purpose of this study. One aspect of swallowing, which the study-specific symptom questions do not fully cover, is the aspect of silent aspiration. In a set of questions on dysphagia symptoms, questions on previous pneumonia events and presence of airway discomfort need to be included. Increased reliability of the VFS examinations could be obtained by performing blinded analysis at a separate occasion from the respective examinations, as well as by having two radiologists evaluating each examination in order to assess inter-judge reliability. However, the assessments of the VFS examinations were performed in collaboration by two professionals, and hence the assessments should be reliable. Our data was collected as patients were followed-up post-treatment at different time points; results in a prospective setting where information on dysphagia is collected prior to treatment may shed further light on the current research question. However, the dispersal in assessment time was assessed in a median split analysis and proved to have negligible impact on the overall results albeit with a trend of stronger correlations for longer follow-up. Finally, our results are based on a relatively healthy and homogenous patient group in terms of treatment intent and comorbidity, which may affect the generalizability of the results.

In conclusion, our data suggest that if a patient reports swallowing difficulties when being asked a direct question, it is likely that he or she also has eating difficulties but not necessarily problems with coughing when eating/drinking. However, if all these three symptoms are reported, it is likely that the patient will present with moderate to severe dysphagia according to PAS and thus should be referred for further evaluation and treatment. The usefulness of these questions as a screening tool for swallowing difficulties needs to be further investigated. However, it is our strong belief that questions on drinking difficulties in this context will be of minor importance, while the remaining three questions investigated in this study will prove to be useful in detecting dysphagia in HNC patients in both outpatient care and inpatient care facilities.
